# Establishment of Japan's First “Podiatry Medicine Center” in a University Hospital

**DOI:** 10.14789/jmj.JMJ21-0031-OT

**Published:** 2022-04-15

**Authors:** RICA TANAKA

**Affiliations:** 1 Center of Podiatry Medicine, Juntendo University Hospital, Tokyo, Japan; 1 Center of Podiatry Medicine, Juntendo University Hospital, Tokyo, Japan; 2 Division of Regenerative Therapy, Juntendo University Graduate School of Medicine, Tokyo, Japan; 2 Division of Regenerative Therapy, Juntendo University Graduate School of Medicine, Tokyo, Japan; 3 Department of Plastic and Reconstructive Surgery, Juntendo University School of Medicine, Tokyo, Japan; 3 Department of Plastic and Reconstructive Surgery, Juntendo University School of Medicine, Tokyo, Japan

**Keywords:** podiatry, podiatry medicine, team medicine, university hospital

## Abstract

Podiatry Medicine is well known medical field in Europe and the United States, which is a discipline that specializes in foot medical care. In Europe and the United States, it is common to see a podiatrist if there are any symptoms in the foot and they provide specialized medical for foot disease. However, in Japan, even if you feel that your foot hurts, there is no specialized medicine to be provided. In Japan, commonly podiatry medicine is often provided by each clinical field or department related to patient's symptoms and there was no university hospital in Japan that advocated Podiatry Medicine. As the aging society progresses in the future, maintaining “walking” is one of the important issues to extend the healthy life span. To overcome this issue and to provide specialized medical care for foot patients in Japan, Juntendo Hospital established the “Podiatry Center” to be the first time in Japan. This center comprehensively assesses the symptoms that occur in the foot, similar to podiatry medicine in Europe and the United States (medical treatment for the lower leg to the foot, not including the knee), and provides multidisciplinary treatment from foot care to cutting-edge medical treatment. The center cooperates with many clinical departments such as plastic and reconstructive surgery, dermatology, vascular surgeon, cardiovascular medicine, diabetes medicine, collagen disease medicine, kidney medicine, orthopedics, rehabilitation department, and nurses, prosthetist, and other professions to carry out diagnosis, treatment, and prevention promptly and effectively for the best for patients with foot diseases.

## Introduction

Podiatry medicine is a field of medicine specialized to study, diagnosis, and treat the disorders of the foot, ankle, and lower extremity. The medicine started in the early 20th century in the United States and now is practiced in United Kingdom, Australia, and Canada. In these countries, podiatrist is a national qualification of a physician who specializes in that practice. There are nine universities that specialize in foot disease and surgery in the United States, and graduates are certified as podiatrists or foot surgeons (national qualification). Currently, in the United States, there are approximately 15,000 podiatrists who diagnose, treat, and provide preventive care for all foot-related illnesses^1)^. The main medical treatments offered by certified podiatrists and podiatric surgeons address a wide range of diseases such as foot swelling, ganglion cyst, Charcot feet, Morton's disease, hallux valgus, shoe problems, tarsal canal syndrome, metatarsophalangeal pain, plantar fasciitis, plantar fibromatosis, athlete's foot, diabetic foot ulcers, and ischemic lower extremities. However, Japan does not have an education or a specialist system for podiatry.

## Current status of foot disease treatment abroad and in Japan

In Japan, depending on the disease, various clinical departments such as plastic surgery, orthopedics, surgery, vascular surgery, cardiovascular medicine, and dermatology provide medical care for patients with a foot disease. However, if team-based and cross-sectional medical care is not provided, it may be difficult to deal with each non-specialized disease. Furthermore, in Japan, many patients ignore foot symptoms despite feeling pain because they do not know which department to visit when the cause is unknown. As society continues to age, maintaining mobility (walking) is one of the most important issues in order to extend healthy life expectancy. We believe that the establishment of a specialized medical care system for foot diseases that enables medical care with appropriate knowledge and technology is indispensable for the future society of Japan.

## Establishing of the Podiatry Center at Juntendo University Hospital

In Japan, other hospitals and university hospitals often treat patients with foot disease in multiple clinical departments (e.g., plastic surgery, dermatology, orthopedics, cardiology, and vascular surgery) under terms such as “foot care outpatient,” “refractory ulcer outpatient,” and “wound care center.”

Presently, it is reported that one foot is amputated every 30 seconds around the world^2)^. Although, medical care for feet in Japan is lagging behind that in Europe and the United States, the Japanese government announced that “prevention of diseases centered on lifestyle-related diseases including cancer” as “Honebuto no Hoshin 2015” which includes medical care for lower limb relief. As a result of the 2016 revision of health and medical service fees, the “additional guidance for peripheral arterial disease in the lower extremities for dialysis patients” was recognized. This was implemented by providing foot care to patients undergoing dialysis and introducing these patients with low blood flow to a medical institution that has a specialized treatment system. Feet are essential in order to maintain mobility (walking), and they need protection to extend an individual's healthy life expectancy^2)^. In the future, as Japan heads toward a super-aging society, establishing a specialized center for foot diseases that can provide appropriate knowledge and advanced technology is needed in order to enhance foot medical care. Therefore, the Podiatry Center was established in Juntendo University Hospital, Japan's first university hospital.

## How do we operate Podiatry Medicine in Japan

**Figure 1:**
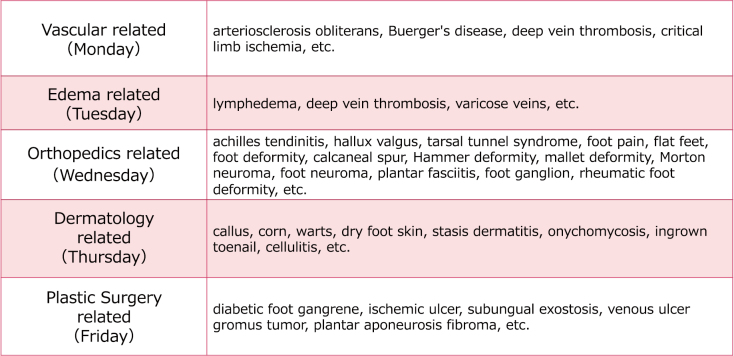
The table is a list of target disease of Juntendo University Hospital Podiatry Center. The disease are seen by a doctor specialized in the field listed in the table.

**Figure 2:**
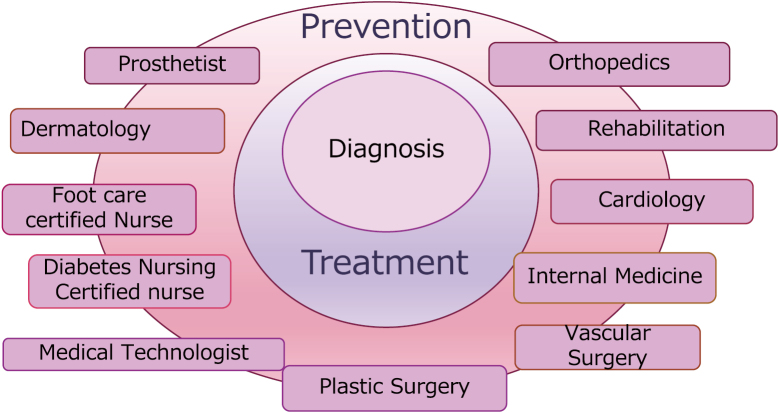
The figure shows the representative members of Podiatry Medicine in Japan which achieved by multidisciplinary team work.

The “Foot Disease Center” provides medical care for diseases handled by American podiatrists (Figure 1). The Podiatry Center brings together specialists in assessment, diagnosis and treatment of foot disease from plastic surgery, orthopedics, cardiovascular medicine, diabetology, nephrology, and rehabilitation and other field of specialists. (Figure 2). Nurse and footcare expert conduct a comprehensive assessment of feet and implements various treatments from foot care to treatment of ingrown nails.

**Figure 3:**
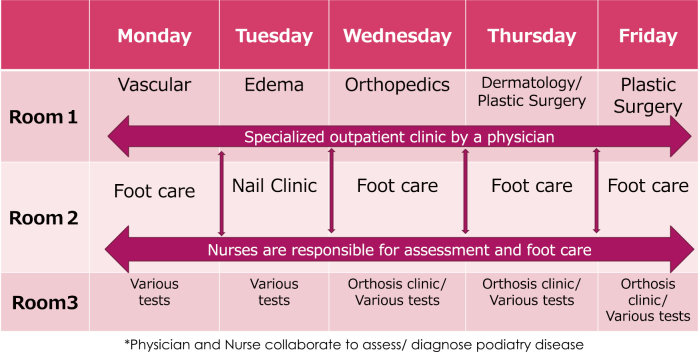
The table show the outpatient clinic responsibilities by the members in the Podiatry Center at Juntendo University Hospital.

**Figure 4:**
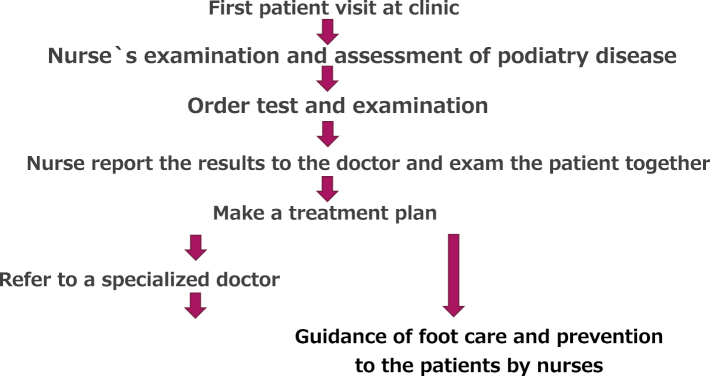
The figure shows the medical examination flow in the Podiatry Center at Juntendo University Hospital processed by the physician and the nurses.

In detail, outpatient clinics are handled by doctors specializing in foot diseases in the fields of cardiovascular medicine and surgery on Monday, vascular surgeon specialized in lymphedema on Tuesday, orthopedics on Wednesday, dermatology on Thursday, and plastic surgery on Thursday evening and Friday. Next to the outpatient clinic in charge of the doctor, a nurses who are certified for foot care instructor will be running the clinic responsible for assessment of podiatry disease and preventive foot care. (Figure 3) The role of the nurse is to comprehensively assess the feet of the first-visited patient and practice treatments such as preventive foot care and ingrown nails. When the first-visit patient receives a medical examination, the nurse conducts a detailed interview and a comprehensive assessment of the feet and reports the contents to the doctor. The doctor requests the necessary tests based on the report of the nurse and proceeds to diagnosis and treatment, but in cases outside the specialty, he or she will refer a specialized doctor in the field (Figure 4). Since podiatry medicine is not a specialized academic field in Japan, most of Japanese doctors in this field are familiar with podiatry disease outside their specific field. Therefore, doctors study at their own interest and augment their knowledge by experience and support by other specialists and nurses.

By sharing the specialized knowledge of foot diseases in each department and combining, examining, and practicing cutting-edge technologies, we will pave the way for cutting-edge medical care (such as regenerative medicine^3)^) from foot care to treatment of intractable diseases. Furthermore, the cooperation between departments will make it possible to provide safe and optimal treatment for diseases that each department has previously dealt with individually.

## Significance and role of the Podiatry Center from the standpoint of plastic surgery

Wound care is an important field of Plastic Surgery. However, there are many foot wound diseases that cannot be treated by plastic surgery alone and require the cooperation of other departments depending on the symptoms. By centralizing the treatment for foot disease, multidisciplinary treatment, which includes cardiovascular medicine, orthopedics, dermatology, rehabilitation, and internal medicine (nephrology, rheumatology, and diabetology) becomes easier to perform. With the cooperation of each department, it will be possible to provide safe and optimal treatment for diseases that each department has previously handled individually. In addition, foot deformities often occur after surgical treatment of foot disease. Continuous care with shoes, orthoses, and foot care is required to prevent the recurrence of foot disease, and we are actively working to prevent recurrence even after treatment is completed^4)^.

Studies have been conducted on how team medical care can avoid lower limb amputation rates for diabetic ulcers, and the results have been reported. Riaz et al reported that prior to team medical care to treat diabetic wounds, lower limb amputation rates decreased from a high baseline of 27.5% to 3.9%^5)^ In Denmark, it was reported that the lower limb amputation rate of 27.2% was reduced to 6.9% for a population of 100,0004, and in the UK, it was reported that it was possible to cause a reduction of lower limb amputation to about 2%^6, 7)^. In the future, I think it will be ideal for us to be involved in the treatment of a wide range of foot diseases that is at par with Western podiatrists without being bound by specialized fields.

## Future direction

There is a wide range of diseases related to foot disease, and in Japan, where podiatry does not exist, there are some diseases for which medical experience and systems are not in place. We wish to contribute to society as a Podiatry Center in Japan's first university hospital that can handle a wide range of patients with foot disease.

## Funding

There are no funding for this manuscript.

## Author contributions

RT was a major contributor in writing the manuscript and approved the final manuscript.

## Conflicts of interest statement

There are not conflicts of interest for this work.
